# COping with Rheumatic Stressors (CORS) questionnaire: validated German translation and cross-cultural adaptation for patients with axSpA

**DOI:** 10.1186/s41687-024-00828-3

**Published:** 2025-01-09

**Authors:** Kristina Vaupel, David Kiefer, Sofia Ramiro, Uta Kiltz, Wim van Lankveld, Ludwig Hammel, Xenofon Baraliakos

**Affiliations:** 1 Clinical Science Scholarship, Harvard Medical School, Boston, MA USA; 2 Ruhr-Universität Bochum, Bochum, Germany; 3 Rheumazentrum Ruhrgebiet, Claudiusstrasse 45, 44649 Herne, Germany; 4 Department of Rheumatology, Leiden University Medical Center, Leiden, The Netherlands; 5 Department of Rheumatology, Zuyderland Medical Center, Heerlen, The Netherlands; 6 Musculoskeletal Rehabilitation Research Group, Institute of Health Studies, HAN University of Applied Sciences, Nijmegen, 6503 GL The Netherlands; 7 Deutsche Vereinigung Morbus Bechterew e.V., Schweinfurt, Germany; 8 RWTH Aachen, Aachen, Germany

**Keywords:** Rheumatic stressors, Pain, Stress response, Coping, Axial spondyloarthritis, Ankylosing spondylitis, Questionnaire, Cross-cultural adaptation, Linguistic validation, Cognitive debriefing

## Abstract

**Background:**

Patients with Rheumatic and Musculoskeletal Diseases, including axial spondyloarthritis (axSpA), may suffer from stressors like pain and functional impairments leading to limitations in their self-perceived health status. The COping with Rheumatic Stressors (CORS) questionnaire was developed to analyze how patients cope with these stressors. The CORS is currently not available in German.

**Objective:**

First, to translate, cross-culturally adapt and to linguistically validate the original Dutch CORS into German. Second, to test the pre-final German translation through cognitive debriefing in patients with axSpA.

**Methodology:**

The original Dutch CORS underwent a multistep cross-cultural adaptation process, as described by Beaton. It was first independently translated into German by bilingual Dutch-German lay and expert translators. Subsequently, it was translated back from the German version into Dutch. Remaining discrepancies were resolved by a scientific committee, resulting in a pre-final German version. This version was then tested through cognitive debriefing by 10 patients with axSpA across a broad spectrum of sociodemographic backgrounds.

**Results:**

Forward and backward translations of the CORS revealed minor discrepancies, mainly based on the degree of formal versus informal language usage, minor semantic errors or unusual syntax, which led to minor modifications in the wording. Reviewed by the scientific committee, the pre-final consensus German version was linguistically validated by cognitive debriefing by 10 patients with axSpA. Cognitive debriefing confirmed and ensured closest linguistic validity for German in Germany and highest equivalence to the Dutch original version.

**Conclusion:**

The German CORS was shown to have high cross-cultural and face validity for the assessment of coping with rheumatic stressors.

**Supplementary Information:**

The online version contains supplementary material available at 10.1186/s41687-024-00828-3.

## Key messages

**•** Cognitive debriefing revealed that the German CORS is highly relevant for patients with axSpA.
**•** The German CORS has high cross-cultural validity to assess coping with rheumatic stressors.
**•** The German CORS has closest linguistic validity and highest equivalence to the Dutch original version

**Supplementary Information:**

The online version contains supplementary material available at 10.1186/s41687-024-00828-3.

## Introduction

Patients with Rheumatic Musculoskeletal Diseases (RMDs) including axial spondyloarthritis (axSpA) may suffer from chronic pain and impaired function, limiting their professional and daily activities as well as their self-perceived health status [1, 2]. AxSpA, including radiographic (r-axSpA), earlier known as ankylosing spondylitis, and non-radiographic (nr-) axSpA, is characterized by inflammation and structural damage in the axial skeleton [1, 3, 4]. Peripheral manifestations (arthritis, enthesitis, dactylitis) and extra-musculoskeletal manifestations (uveitis, psoriasis, inflammatory bowel disease) may occur in the course of the disease [1]. Both, inflammation and structural damage lead to reduced social interactions and impaired well-being [5] resulting in an overall impaired health-related quality of life (HRQoL) [6]. In addition to the burden of the rheumatic disease itself, patients also suffer from the burden of comorbidities like cardiovascular diseases, osteoporosis, depression and fibromyalgia [7–9]. All these burdens are chronic stressors and might interrelate with pain, physical function, dependency and work related issues (work participation; workplace instability and unemployment; (temporary) work incapacity and disability) [10–12]. Interpersonal stressors comprising emotionally stressful episodes (verbal and non-verbal conflicts); negative attitudes, behavior or feelings like social rejection/isolation [13] were investigated in patients with RMDs, predicting a higher disease activity under the influence of interpersonal stressors [14], thereby increasing the comorbidity burden through an elevated risk for depression [15, 16]. In summary, the understanding of rheumatic stressors and the individual’s daily stress response provide patients with insights into their self-perceived health status and empowers them to self-report the respective outcome [17, 18].

Stress responses such as coping behavior, encompassing avoidance-, resistance-, resilient/adaptive- or denial-coping, are therefore crucial for treating physicians to understand the individual patient journey [19–23]. For the avoidance coping style it was revealed that it worsens the functional outcome [25]. Coping itself is defined as a continuous process between the stressor and the individual reaction to stress with constantly changing cognitive and behavioral efforts to manage specific external and/or internal demands [24, 25]. Thus, coping comprises two dimensions, cognitive coping through emotions, thoughts, perceptions, cognitive strategies and the planning process of coping as well as behavioural coping enabeling actions in stressful situations [26]. Studies on coping strategies in patients with axSpA revealed various facets of individual coping behavior [24, 27, 28]: the influence of illness duration and pain intensity [24], the impact of illness perceptions and coping on the relationship between back pain and health outcomes [29], and the persistence of illness perception and coping strategies over time [30].

First analyses of the coping capacity for rheumatic stressors in patients with RMDs have been performed with the COping with Rheumatic Stressors (CORS) questionnaire, initially developed for patients with rheumatoid arthritis [28, 31, 32]: instead of other pain-focused instruments [33–35], the CORS concentrates on analyzing the three most pivotal rheumatic stressors: pain, limitations, and dependency [28, 31, 32]. After no coping assessment for rheumatic stressors was available for patients with axSpA, the CORS was adapted for axSpA and at first cross-culturally adapted for Spanish and Turkish patients [36, 37]. Thus, since the CORS questionnaire is currently not available in German, this study aims at first translating and cross-culturally adapting the Dutch CORS into German and second, to field-test the questionnaire with German patients with axSpA.

## Methods

For the translation and cross-cultural adaptation of the CORS into German, and instead of applying linguistic validation methods following the ISPOR principles, using professional translators, AI-enabled machine translation or neuro-linguistic programming which were adopted as industry best practices and accepted by regulatory bodies (FDA; EMA), this translation follows the Assessment of SpondyloArthritis international Society (ASAS) handbook and the therein introduced principles for translation, cross-cultural linguistic adaptation and validation as outlined by Beaton et al. [38, 39]. Ethics approval for the entire study was granted by the ethics committee of the Ruhr-Universität Bochum, Germany (Register No. 21-7179).

### CORS questionnaire

The CORS questionnaire is designed to assess coping strategies specific to arthritis and derived from patients themselves [28]. It evaluates eight coping strategies, focusing on the primary chronic stressors associated with inflammatory rheumatic disease, such as pain, limitations, and dependency in individual scales. The coping strategies of “decreasing activities to cope with pain” and “pacing to cope with limitations” are classified as behavioral coping strategies, while the other six coping strategies are considered cognitive.

**Fig. 1:**
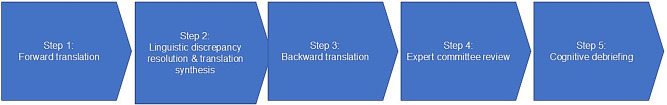
Cross-cultural adaptation process

*Pain-related coping strategies* are measured through three scales (three coping styles): comforting cognitions (nine items), decreasing activities (eight items), and diverting attention (eight items), resulting in 25 items within the total pain scale. *Coping with limitations* is assessed through three scales (three coping styles): optimism (five items), pacing (adapting one’s activity level, ten items), and creative solution seeking (eight items), resulting in 23 items of the entire coping with limitations scale. *Coping with dependency* is addressed by two scales (two coping styles): accepting one’s level of dependency (six items) and showing consideration (seven items), in total 13 items for the total dependency cluster of coping strategies. Each item requires patients to indicate the frequency of their use of a particular coping strategy using a four-point scale (1 = seldom or never, 2 = sometimes, 3 = often, 4 = very often). Scale scores are calculated by summing the scores of individual items, with higher scores indicating more frequent use of a specific coping style [23, 36]. Thus, the total cluster of pain coping strategies score ranges from 25 to 100, all limitations items from 23 to 92 and coping with dependency ranges from 13 to 52 (Fig. 1).

### Cross-cultural adaptation

The entire cross-cultural adaptation process was conducted following the methodology proposed by Beaton (Figure S1) [38]:

**Step 1: Forward translation.** For each tramslation, the forward (step 1) and backward translation (step 2), two native bilingual Dutch/German axSpA patients were recruited with the help of the German patient organization “Deutsche Vereinigung Morbus Bechterew e.V.”.

In step 1, two patient translators (one informed about the content of cross-cultural adaptation and linguistic validation due to three participations in such projects; one uninformed) independently translated the Dutch original source version into German, the target language.

In contrast to the content knowledge of the informed patient translator about cross-cultural adaptations and linguistic validations, the uninformed patient translator has never before participated in cross-cultural adaptation and linguistic validation projects and thus, provided the layman’s perspective, highlighting ambiguous meanings in the original questionnaire [38, 40]. Each translator produced a written report of the translation with additional comments to highlight discrepancies, challenging phrases or uncertainties. Discrepancies between the translations were resolved by the translators and two experts (Step 4: Expert committee).

**Step 2: Synthesis of the translations and consensus formation.** The two translators and a recording observer together synthesized the results of the translations, mainly by resolving linguistic discrepancies.

**Step 3: Backward translation.** Two independent lay translators, with no prior knowledge of the CORS questionnaire, translated the common consensus backward, i.e., the initial translation (T1 + 2) back into two separate Dutch versions. These backward translators were unfamiliar with the CORS outcome measurement instrument, had no medical background, and were new to cross-cultural adaptations. This approach ensured content validity, minimized information bias, and uncovered unclear wording, inconsistencies, and conceptual flaws in the translations.

**Step 4: Expert committee review.** A review board was formed to review all reports and translations and to achieve consensus on all aspects of the translations, including items, instructions, and response options. Therefore, two expert participants acted as recording observer and methodologist, one rheumatologist and one non-medical researcher, were responsible for the separate translation processes: one for the forward and the other for the backward translation.

**Step 5: Cognitive debriefing.** In accordance with the approach taken by the Spanish translation, a group of ten patients diagnosed with axSpA was recruited for the cognitive debriefing of the prefinal consensus version.

In addition to the results of the translations and the cognitive debriefing of the pre-final consensus German CORS version, various outcome measures, including pain, C-reactive protein (CRP), erythrocyte sedimentation rate (ESR), and disease activity (Axial Spondyloarthritis Disease Activity Score, ASDAS; ASDAS with ESR, ASDAS-ESR; Bath Ankylosing Spondylitis Disease Activity Index, BASDAI), functional status (Bath Ankylosing Spondylitis Functional Index, BASFI), overall functioning and health (Short Form 36, SF36; Assessment of Spondyloarthritis International Society Health Index, ASAS HI), modified-Short QUestionnaire to Assess Health-enhancing physical activity (mSQUASH) and CORS, were assessed to characterize the patient group. These patients represented a diverse group of patients with axSpA in terms of demographic and clinical data (Tables 1 and 2).

**Table 1:** Demographic data from individual patients of the cognitive debriefing

Patient	Age*	Sex*	BMI	Employment status*	Employment duration (years)	Weekly working hours	Highest education degree*
1	43.2	w	32.5	3	16	54.0	2
2	53.2	m	26.6	1	36	40.0	2
3	56.2	m	25.4	3	36	40.0	2
4	50.0	m	25.9	1	35	37.5	2
5	35.4	m	23.5	1	1	40.0	4
6	52.8	w	32.9	2	37	18.0	1
7	42.3	w	24.2	1	12	40.0	4
8	63.0	m	24.6	3	47	40.0	2
9	55.8	m	27.1	1	38	50.0	2
10	59.6	m	19.8	3	26	40.0	2
Mean (SD)	51.2 (8.5)	30% Women	26.3 (4.0)	-	28.4 (14.3)	37.6 (10.5)	-

*Results as mean values (SD, standard deviation); sex: m: man sex: w, woman; BMI: Body Mass Index; employment status: 0: jobseeker, 1: full-time, 2: part-time (incl. part-time due to illness), 3: retired/early retirement due to permanent illness; highest level of education: 0 = primary school, 1 = intermediate school leaving certificate/secondary school leaving certificate, 2 = university entrance qualification/vocational training, 3 = university degree < 4 years, 4 = university degree > 4 years

**Table 2:** Clinical data from individual patients of the cognitive debriefing

Patient	r-/nr axSpA	HLA-B27*	Symptom duration (years)	Pain NRS (0–10)	CRP (mg/dl)	ASDAS-CRP	BASDAI (0–10)	BASFI (0–10)	SF36 (%)		ASAS HI(0–17)
									MCS (0-100)	PCS (0-100)	
1	0	pos.	7.3	1.0	0.1	3.5	3.03	9.30	29.0	4.0	12.0
2	0	neg.	15.3	7.0	0.2	2.5	5.25	5.90	78.3	17.5	10.0
3	1	pos.	5.3	7.5	0.2	1.7	3.05	4.20	59.4	15.0	11.0
4	0	neg.	8.1	7.0	2.2	4.1	3.60	0.80	76.4	65.0	3.0
5	0	pos.	6.1	7.0	0.1	2.4	3.80	3.40	77.8	63.8	6.0
6	0	pos.	5.1	7.5	0.6	3.5	6.81	5.50	28.8	31.6	12.0
7	0	pos	7.1	6.0	0.1	2.0	4.33	4.40	50.0	75.0	9.0
8	1	pos.	34.2	8.0	0.1	3.1	4.73	8.40	66.6	36.9	10.0
9	1	pos.	23.2	7.0	1.4	3.6	5.28	5.40	76.1	23.8	8.0
10	1	neg.	25.6	7.0	0.0	3.6	9.65	9.10	24.5	21.9	13.0
Mean (SD)	40%r-axSpA	70% HLA-B27 pos.	13.7 (10.4)	6.5 (2.0)	0.5 (0.7)	3.0 (0.8)	4.95 (2.02)	5.64 (2.69)	56.7 (13.7)	35.5 (20.4)	8.7 (3.1)

*Results as mean values (SD, standard deviation); abbreviations (from right to left): r-axSpA: radiographic axSpA = 0; nr-axSpA: non-radiographic axSpA = 1; HLA-B27: human leukocyte antigen B27: 0: negative, 1: pos; NRS: numerical rating scale, 0 = no pain, 10 = worst pain; CRP: C-reactive protein; ASDAS: Axial Spondyloarthritis Disease Activity Score; BASDAI: Bath Ankylosing Spondylitis Disease Activity Index; BASFI: Bath Ankylosing Spondylitis Functional Index; SF36: Short Form 36; MCS: Mental Component Score; PCS: Physical Component Score; ASAS HI: Assessment for Ankylosing Spondylitis Society Health Index; mSQUASH: modified-Short QUestionnaire to Assess Health-enhancing physical activity

Ten patients with axSpA and German origin were asked to complete the German CORS and were subsequently invited to participate in face-to-face in-depth interviews with a physician for cognitive debriefing. Each participant was questioned about quality criteria such as comprehensibility, understandability, acceptability, and clarity of the response options of all items in each domain in order to ensure the German translation, cross-cultural adaptation and validation for Germany. Therefore, throughout all items per domain, the total number of queries per domain for each criterion was recorded from each participant (Supplementary Table 3). Based on the queries per criterion, the queries per item and the average number of queries per participants were calculated.

The cognitive debriefing served several purposes: [1] to assess the level of comprehension and understanding of the consensus version of the translation among individuals diagnosed with axSpA [2], to determine if German patients understood the translated questionnaire to the same degree as the original, and [3] to ensure that all four levels of equivalence to the Dutch original were met (semantic, idiomatic, experiential, and conceptual equivalence).

During the interviews, patients were given ample time to ask questions, provide comments on the questionnaire, and request explanations if an item, its meaning or the response option was not fully understood by the patient. Additionally, all comments regarding the questionnaire were recorded, and if an item or a response option in the German CORS raised questions, the meaning of conflicting items was thoroughly discussed and evaluated for potential modifications or changes.

## Results

### Forward-translation

Both translators returned their independently translated questionnaire with comments. Discrepancies between both translations were mainly based on the degree of formal language usage versus informal, but easy to understand language usage such as lay terminology or Leichte Sprache [41]. Reviewing of their returned translations revealed additional differences (Supplementary Table S1):

(A) In the introduction to the questionnaire, “beschreven gedrag” was translated to “beschriebenen Fall” or further down, “gedrag” was translated to “Beitrag” or, in the introduction to section “Coping with Limitations”, to “Auftrag”. In order to resolve the inconsistent translation of the Dutch word “gedrag”, it was consistently translated to “Verhaltensweisen” after expert discussion.
(B) In section “Coping with Pain”, item 2 ”Ik houd op met mijn bezigheden.” was translated to “Ich stelle meine Bemühungen ein.”. Compared to the Dutch original version, this item was corrected to “Ich stelle meine Aktivitäten (“bezigheden”) ein.”, after discussion with the translators (Step 1) and review by the expert committee (Step 4).

Additionally, within the context of the entire pain statement, in item 10, the phrase “niet stil te blijven staan” (”nicht still zu halten”) was translated to “sich nicht damit zu beschäftigen”. Thus, “Ik probeer niet bij de pijn stil te blijven staan.” was translated to “Ich versuche, mich nicht mit dem Schmerz zu beschäftigen.”.

Furthermore, item 25 “Ik probeer de moed erin te houden.” was first translated to “Ich versuche mutig zu bleiben.” (“I try to keep courage.”), but after discussion of this too literal translation and unusual German phrase, the item was corrected to “Ich versuche den Mut nicht zu verlieren.” (“I try not to lose courage.”) which is a common German phrase.

(C) In section ”Coping with Limitations”, minor discrepancies such as the selection of common German phrases, the selection of verbs and their respective prepositions were resolved as in item 5: “Ik hou rekening met m’n beperkingen.”, translated to “Ich beschäftige mich mit meinen Einschränkungen.” was corrected to “Ich berücksichtige meine Einschränkungen.”.
(D) In section ”Coping with Dependence”, another semantic error was detected, resulting from word-wise, literal translation which led to errors in meaning and logic: item 10, “ontzien” was translated to “fernhalten/keep other people away”, but did not fit into the sentence’s context. Thus, item 10 was later changed to “Ich versuche andere zu schonen.“.

Corrections were also applied if the chosen wording had an unusual German syntax such as in the given example “Ich gehe in die Dusche.” instead of “Ich gehe duschen.” or if no common agreement about the meaning of a sentence could be reached. Finally, for each item, discrepancies were solved, and a consensus preliminary German version was drafted.

### Backward translation

Comparing the backward translated Dutch version to the Dutch original version, only minor differences were detected because in the intermediate step of the forward translation to German, a good consensus version was achieved.

Some items like item 18 of the pain item set also revealed incremental translational adaptations: From the original Dutch version “Ik neem iets onder handen.” (”Ich nehme etwas in die Hand.“), the German contextual phrase “Ich unternehme etwas.” (”I do/undertake something.”) was derived which in the Dutch back translation led to “Ik doe iets.” (”I do something.”).

In the consensus finding process through the expert review, items also became more intentional and targeted: ”waardoor” in the Dutch original version of pain cluster item 24 (“Ik ga iets doen *waardoor* ik de pijn niet voel.”) was translated with “wodurch” in the German forward translation (”Ich mache etwas, *wodurch* ich den Schmerz nicht fühle.”). In the subsequent backwards translation of ”wodurch” to Dutch, it became “zodat” (“sodass”/”so that”) (“Ik zal iets doen *zodat* ik de pijn niet voel.”).

In the final expert review, the translation ended in the intentional “damit” (“Ich mache etwas, damit ich den Schmerz nicht fühle.”) to underline that the patient explicitly does something to at least distract himself from pain or to prevent the feeling of pain at all. The expert review committee and the translators agreed that the back translation presents an equivalent version of the original version on all semantic and linguistic levels as well as in its conceptual meaning.

### Cognitive debriefing

For the field testing of the preliminary German CORS version, 10 Patients were enrolled when diagnosed with axSpA (4 with r-axSpA; 6 with nr-axSpA) according to the rheumatologist and were bilingual native in German and in Dutch. The group of patients was heterogenous in age, sex, HLA-B27/genetic background, disease duration, educational background and other sociodemographic data (Tables 1 and 2).

All patients read the instructions of the questionnaire before they started to fill it in, and a second time before answering the individual question. During the interview, patients were given unlimited time to ask questions, comment on the questionnaire, and request explanations. Queries per item and domain (Supplementary Table S2), comments and questions on the questionnaire were recorded: Thereof, coping with pain comprised queries in item 8, 10, 18 and 19, the limitations domain contains queries regarding items 2, 4, 8, 12, 17, 18, 19 and 21. Additionally, queries in the dependency domain affects items 2, 3, 5–7, 10, 11 and 13 (all in Supplementary Table S2). On average, patients took 16,7 min to fill in the questionnaire.

In general, all patients positively perceived the CORS questionnaire, felt understood by the questions that were being asked, especially through the main three clusters of rheumatic stressors, pain, limitations and dependency which, according to the patients, exatly captured their most stressful symptom areas. Therefore, all patients considered the CORS questionnaire relevant.

However, as a result of the cognitive debriefing, patients stated that questions were not easy to answer, since they were too general (items with the term “situation” and regarding “optimism”), some items did not fit to the individual disease situation and thus, were not self-explanatory to the patient, leading to discussions and required explanations. Especially in the pain domain, items 2 and 3 as well as items 10, 18, 19; in the limitation set, items 12, 17, 19, 21 and in the dependency set, items 3, 5, 10, 11 and 13 were mentioned by 7 (70%) patients as redundant.

Six patients (60%) also mentioned that the CORS questionnaire item set alone is not sufficient to assess how the patient is coping with the disease and that other measures (pain scale, disease activity) should be integrated in the questionnaire or at least also be part of the interview.

Evaluation of the queries by descriptive statistics (Supplementary Table 3) revealed that for the pain domain and the criterion “comprehensibility”, eight queries were recorded, indicating that 32% of the 25 items were difficult to comprehend, but were less difficult to understand (24%), fully accepted (0 queries) and were 100% clear in the response option. On average and among all four criteria, for the entire pain domain, 14 queries were recorded, equivalent to 23% of total queries for the pain domain. Both domains, “limitations” and “dependency” revealed higher query numbers regarding comprehensibility (40% limitations domain, 61,5% dependency domain), but like in the pain domain, items were less difficult to understand (30,4% limitations domain; 30,8% dependency domain), fully accepted (0 queriesfor both limitations and dependency domain) and were both, limitations and dependency domain 100% clear in the response option. Since all items in all three domains did not miss clarity in the perceived response option, this result re-ensures the quality of the forward-backward translation procedure, in which minor modifications were applied resolving those discrepancies prior to the cognitive debriefing. Thus, the wording of the response options was not required to change after the cognitive debriefing.

The final German CORS questionnaire was developed by an expert review committee after resolving the queries from the cognitive debriefing (Supplementary Table S2 and S3) and is presented in Supplementary Figure S1.

## Discussion

The original Dutch CORS questionnaire was translated, culturally adapted and validated in German according to the method described Beaton et al. [38, 42]. Translations, forward and then backward, showed only slight modifications to the Dutch original and thus, ensured high language equivalence with simultaneous language fidelity in conveying both, information and culture-specific terms. Overall, the interviewed patients found the questionnaire applicable and relevant. This linguistic and cultural adaptation supports the German CORS questionnaire’s suitability for assessing coping strategies in German-speaking patients with axSpA.

The development of coping mechanisms plays a pivotal role in the effective management of RMDs including axSpA since they are significantly influenced by patients’ beliefs and their understanding of their specific medical condition. Coping strategies may help in reducing, mastering, minimizing, or tolerating pain [30, 43]. Consequently, it is essential to evaluate these coping strategies comprehensively for a holistic approach to disease management. While coping is often perceived as a personal trait, it is substantially molded by external factors. Therefore, individuals tend to develop tailored coping mechanisms in response to specific circumstances, which, in turn, can significantly impact their perception of the disease [44].

Notably, in patients with axSpA, certain coping strategies, such as decreasing the level of physical activity following back pain, have been shown to have a negative effect on their quality of life [29]. The use of avoidant coping strategies seems to be primarily influenced by physical function and, to a lesser extent, pain [23]. Furthermore, it has been demonstrated that coping strategies remain remarkable stable among patients with axSpA over time [23, 30].

The manner in which patients cope with pain and limitations has been found to be closely associated with their overall well-being, underscoring the clinical significance of coping strategies in the management of RMDs [28, 31, 32]. Furthermore, the impact of patient education on stressors, individual motivation to understand their medical condition, and the completion of medical forms for coping strategies warrants more in-depth investigation.

Promoting active coping behaviors through education and patient support in individuals with RMDs may not only enhance their perceived social support but also improve their overall quality of life [45]. This highlights the potential of patient education to positively influence coping strategies and, consequently, patient outcomes.

Despite the potential advantages of interventions aimed at enhancing coping strategies, there is a notable scarcity of studies that specifically evaluate coping in the context of RMDs. One contributing factor to this gap in the literature may be the limited availability of suitable instruments for measuring coping [28]. Consequently, there is a need for robust coping measurement instruments tailored to the unique challenges posed by RMDs. So far, coping measurements are not included in domains of the Assessment of SpondyloArthritis international Society-Outcomes Measures in Rheumatology (ASAS-OMERACT) core domain set for axSpA [46, 47]. Assessing coping mechanisms can be achieved through the use of Patient-Reported Outcomes (PROs), which provide valuable insights into disease outcomes from the patient’s perspective [48]. In terms of patients with axSpA, PROs are routinely employed to evaluate various aspects such as health status, functional capacity, and disease activity. The CORS questionnaire holds a unique advantage as it directly addresses coping styles that are closely related to RMD-specific stressors [31, 32].

Therefore, the CORS questionnaire fills a critical gap by providing a comprehensive tool to evaluate coping strategies that are highly relevant for patients with axSpA. This instrument allows for a more nuanced and condition-specific assessment of coping mechanisms, acknowledging the diverse range of cognitive and behavioral responses that may manifest in response to the challenges posed by axSpA. Moreover, through translation and cross-cultural adaptation in multiple languages, the CORS questionnaire has the potential to become a widely used international assessment tool for clinical trials in axSpA, allowing for cross-linguistic comparisons and enhancing its utility beyond language barriers [36, 37].

Whether this underaveraged coping response to dependency is patient- or stressor-dependent, remains to be elucidated. From all identified coping styles and behaviors such as avoidance coping [23], there has to be a common expert understandig which coping behavior in reaction to which stressor is not only (under) developed, but most appropriate and beneficial for the patient.

The most appropriate coping strategy for each rheumatic stressor could thus be selectively reinforced by behavioral therapy (rheumatic stressor targeted behavioural therapy) in order to enhance coping flexibility and to prevent stress-related (mal-) adjustments [49]. In the long term perspective, the CORS questionnaire has to prove whether this instrument could be used to track and steadily improve coping behaviour, stabilize enhance coping flexibility and foster overall coping efficacy. To implement the German CORS version for the coping assessment of patients with axSpA for both, clinical practice and research, additional testing must follow: The retention of the psychometric properties (validity, internal consistency, sensitivity) must be analyzed as well as the questionnaire`s test-retest reliability and validity in a larger patient population in order to prove that the final German CORS demonstrated the measurement properties needed for the intended application [38, 50].

This study has some limitations: At first, all participants were from Germany, which gives good content validity for German as spoken in Germany. Linguistic differences with the five other German speaking countries Switzerland, Austria, Lichtenstein, Belgium and Luxembourg were not taken into account. Hence, with the presented German CORS, cross-cultural adaptation and validation to those countries is not ensured. However, in these five countries, German in the so-called Hochdeutsch or Standarddeutsch is the official written and spoken language [51]. Since the translated German CORS questionnaire contains only Hoch- or Standarddeutsch and does not include national or regional varieties or dialects, based on our experience with translations, debriefings, cross-cultural adaptations and validations into German (meaning Hoch- or Standarddeutsch), we cannot identify language obstacles inhibiting German-speaking patients and rheumatologists from Austria, Switzerland, Belgium, Luxembourg and Liechtenstein from understanding the German CORS version.

Another limitation was the small number of participants of the cognitive debriefing. We decided to only interview 10 patients as representatives for axSpA as other translations of PROs in the validated for axSpA were also using similar numbers of patients [36, 37, 52, 53].

## Conclusion

The participants in the cognitive debriefing process found the final German version of the CORS questionnaire highly relevant for understanding their personal approaches to coping with stress. Additionally, scientific reviews ensured the highest linguistic and conceptual equivalence to the Dutch original, confirming its validity throughout the translation and cross-cultural adaptation. This robust adaptation process underscores the German CORS as a valuable tool for both clinical assessment and research on coping strategies in axSpA.

## Electronic Supplementary Material

Below is the link to the electronic supplementary material.

Supplementary Material 1

## Data Availability

Data and material are available upon reasonable request from the corresponding author.

## Abbreviations

ASAS HI: Assessment of Spondyloarthritis International Society Health Index
AxSpA: Axial SpondyloArthritis
ASDAS: Axial Spondyloarthritis Disease Activity Score
ASDAS-ESR: Axial Spondyloarthritis Disease Activity Score with Erythrocyte Sedimentation Rate
BASDAI: Bath Ankylosing Spondylitis Disease Activity Index
BASFI: Bath Ankylosing Spondylitis Functional Index
BMI: Body Mass Index
CORS: COping with Rheumatic Stressors
CRP: C-Reactive Protein
ESR: Erythrocyte Sedimentation Rate
HLA-B27: Human leukocyte antigen B27
HRQoL: Health-Related Quality of Life
M: Man/male
MCS: Mental Component Score
mSQUASH: Modified-Short QUestionnaire to Assess Health-enhancing physical activity
nr-axSpA: Non-radiographic axSpA
NRS: Numerical rating scale
OMERACT: initially “Outcome MEasures in Rheumatoid Arthritis Clinical Trials”, now “Outcome Measures in Rheumatology”
PCS: Physical Component Score
PROs: Patient-Reported Outcomes
r-axSpA: Radiographic axSpA
RMDs: Rheumatic Musculoskeletal Diseases
SD: Standard Deviation
SF36: Short Form 36
W: Woman/female
