# Supplementary material for: COping with Rheumatic Stressors (CORS) questionnaire: validated German translation and cross-cultural adaptation for patients with axSpA
Source: J Patient Rep Outcomes. 2025 Jan 9;9:5. doi: 10.1186/s41687-024-00828-3 (PMC11718023; doi:10.1186/s41687-024-00828-3)
Supplement: Supplementary file 1 — Supplementary Material 1 [file 41687_2024_828_MOESM1_ESM.docx]

**Supplement**

**1. Supplementary Tables**

**1.1 Supplementary Table S1: Forward-translation of the original Dutch CORS questionnaire into German – Queries**

**1.1.1 Coping with pain**

**1.1.2 Coping with limitations**

**1.1.3 Coping with dependency**

**1.2 Supplementary Table S2: Cognitive debriefing queries and decisions from the expert-committee**

**1.2.1 Coping with pain**

**1.2.2 Coping with limitations**

**1.1.3 Coping with dependency**

**1.3 Supplementary Table S3: Descriptive statistics of the cognitive debriefing results**

**2. Supplementary Figure S1: Final German CORS version**

**1. Supplementary Tables**

1.1 Supplementary Table S1: Forward-translation of the original Dutch CORS questionnaire into German – Queries

**1.1.1 Coping with pain**

| **Original Dutch Item** | **Consensus German forward translation^[[1]](#footnote-1)^** | **Queries** | **Consensus version for backward translation German 🡪 Dutch** |
| --- | --- | --- | --- |
| **Omgaan met pijn:** | **Umgang mit Schmerzen:** | The Dutch word “gedrag” was at three different introductory positions (for detailed reference, see results part) translated into the German words “Fall”, “Beitrag” or “Auftrag” In order to resolve this discrepancy, after expert discussion, “gedrag” was consistently translated to “Verhaltensweisen”: | Verhaltensweisen |
| 2. Ik houd op met mijn bezigheden. | Ich stelle meine Bemühungen ein. |  | Ich stelle meine Aktivitäten ein. |
| 5. Ik beperk me tot eenvoudige bezigheden. | Ich beschäftige mich mit einfachen Dingen. |  | Ich beschränke mich auf einfache Tätigkeiten. |
| 10. Ik probeer niet bij de pijn stil te blijven staan. | Ich versuche, mich nicht ununterbrochen mit den Schmerzen zu beschäftigen. | “Ununterbrochen“ was proposed to deleted | Ich versuche mich nicht mit dem Schmerz zu beschäftigen. |
| 15. Ik ga iets doen wat ik plezierig vind. | Ich mache etwas, was ich schön finde. |  | Ich mache etwas, das ich schön finde. |
| 16. Ik probeer optimistisch te blijven. | Ich probiere, optimistisch zu bleiben. | The Dutch verb “probeer“ was consistently translated to the German verb “versuchen”. | Ich versuche optimistisch zu bleiben. |
| 17. Ik zorg dat ik me niet lichamelijk hoef in te spannen. | Ich versuche, dass ich mich körperlich nicht damit beschäftige. | Wording was changed. | Ich versuche mich körperlich nicht anzustrengen. |
| 19. Ik probeer niet aan de pijn te denken. | Ich probiere nicht an die Schmerzen zu denken. | The Dutch verb “probeer“ was consistently translated to the German verb “versuchen”. | Ich versuche nicht an die Schmerzen zu denken. |
| 25. Ik probeer de moed erin te houden. | Ich versuche mutig zu bleiben. | Additionally, item 25 ”Ik probeer de moed erin te houden.” was first translated to ”Ich versuche mutig zu bleiben.” (”I try to keep courage.”), but after discussion, the item was corrected to ”Ich versuche den Mut nicht zu verlieren.”. | Ich versuche, den Mut nicht zu verlieren. |

**1.1.2. Coping with limitations**

| **Original Dutch Item** | **Consensus German forward translation** | **Queries** | **Consensus version for backward translation German 🡪 Dutch** |
| --- | --- | --- | --- |
| **Omgaan met beperkingen** | **Umgang mit Einschränkungen** |  |  |
| 1. Ik stel me tevreden met wat ik wél kan. | Ich bin zufrieden mit dem, was ich wohl kann. | Wording was changed. | Ich bin zufrieden mit dem, was ich tun kann. |
| 4. Ik probeer de positieve kanten van de situatie te zien. | Ich versuche, die positiven Seiten von der Situation zu sehen. | Wording was changed. | Ich versuche, die positiven Seiten der Situation zu sehen. |
| 5. Ik houd rekening met m'n beperkingen. | Ich beschäftige mich mit meinen Behinderungen. | In the english and in the spanish translations, the original dutch word “beperkingen” was translated to “limitations” or “limitaiones“. After expert discussion, the translation of “beperkingen” into the German “Behinderung” is more far-reaching and serious for the patient. In a patient, especially in the early axSpA stage, the word "disability" - even if it is certainly diagnostically correct - may trigger fears of loss of joint function, enhanced disease progression and loss of mobility and independence. Furthermore, not only can fear be caused by inconsiderate, unempathetic wording, but also the loss of hope, motivation, therapy adherence ("It's no use anyway.") and trust in the strength in the own body are to be feared. | Ich berücksichtige meine Einschränkungen. |
| 6. Ik zoek oplossingen voor mijn beperkingen. | Ich suche nach Lösungen für meine Behinderung. | „Behinderung“ was proposed to change to „Einschränkungen“. | Ich suche nach Lösungen für meine Einschränkungen. |
| 8. Wat ik moet doen, doe ik op een aangepaste manier. | Was ich tue, tue ich angepasst. | Wording was changed. | Was ich zu tun habe, tue ich auf eine angepasste Weise. |
| 10. Ik probeer optimistisch te blijven. | Ich probiere optimistisch zu bleiben. | The Dutch verb „probeer“ was consistently translated to the German verb “versuchen”. | Ich versuche optimistisch zu bleiben. |
| 11. Ik neem de tijd voor mijn bezigheden. | Ich nehme mir Zeit für meine Beschäftigungen. | Wording was changed. | Ich nehme mir Zeit für meine Aktivitäten. |
| 12. Ik denk na over manieren waarop ik dingen toch kan doen. | Ich denke nach, wie ich einige Dinge doch tun kann. | Perceived redundancy with items. | Ich überlege wie ich die Dinge doch tun kann. |
| 13. Ik probeer vertrouwen in de toekomst te hebben. | Ich probiere, Vertrauen in die Zukunft zu haben. | The Dutch verb „probeer“ was consistently translated to the German verb “versuchen”. | Ich versuche, Vertrauen in die Zukunft zu haben. |
| 17. Ik zoek een manier om iets tóch te kunnen. | Ich versuche, doch etwas zu können. | Since it is not “test” or a trial in which something is tried or tested, the patients´ translations of the phrase “Ik zoek een manier” into the German phrase “ich versuche” was corrected into “Ich suche nach einer Möglichkeit“. | Ich suche nach einer Möglichkeit, doch etwas tun zu können. |
| 19. Ik bedenk nieuwe manieren om de zaken aan te pakken. | Ich denke nach neuen Möglichkeiten, um die Dinge anzufangen. |  | Ich denke mir neue Möglichkeiten aus, um die Dinge anzugehen. |
| 20. Ik rust op tijd uit. | Ich ruhe mich zur richtigen Zeit aus. | As also in item 22, „op tijd“ is translated to „zur richtigen Zeit”, but is misleading and therefore was corrected to “beizeiten” (item 20) or ”rechtzeitig” (item 22). | Ich ruhe mich beizeiten aus. |

**1.1.3. Coping with dependency**

| **Original Dutch Item** | **Consensus German forward translation** | **Queries** | **Consensus version for backward translation German 🡪 Dutch** |
| --- | --- | --- | --- |
| **Omgaan met afhankelijkheid:** | **Umgang mit Abhänglichkeit** | Since the German translation of “afhankelijkheid“ into “Abhänglichkeit“ is uncommon, it was changed into “Abhängigkeit” (items 3, 5, 7, 9, 11). | Umgang mit Abhängigkeit |
| 3. Ik probeer in mijn afhankelijkheid te berusten. | Ich versuche, mich in meiner Abhänglichkeit zu verstehen. | According to the experts, these items does not center around the own self-imagination, but rather about resigning oneself to the own perceived dependency. Thus, “te berusten” was corrected into “abzufinden”. | Ich versuche, mich mit meiner Abhängigkeit abzufinden. |
| 8. Ik pas me aan anderen aan. | Ich passe mich an andere an. | Wording was changed. | Ich passe mich anderen an. |
| 9. Ik probeer me niet druk te maken over mijn afhankelijkheid. | Ich versuche keinen Stress zu haben mit meiner Abhänglichkeit. | In this case, the literal translation of “druk te maken” into “unter Druck zu setzen” as in the consensus version for backward translation German to Dutch after expert discussion, would have been closer to the intended meaning than the first German translation “keinen Stress zu haben”, which is also too colloquial. | Ich versuche, mich mit meiner Abhängigkeit nicht unter Druck zu setzen. |
| 10. Ik probeer anderen te ontzien. | Ich versuche, andere fernzuhalten. | At first, “ontzien“ was translated with “to keep others away”, but since “ontzien” has a more caring meaning – the Spanish version (36) translated “anderen te ontzien” with “not to be a burden for others” – it was translated to the German verb “schonen”. | Ich versuche, andere zu schonen. |
| 13. Ik probeer niet teveel te vragen aan één persoon. | Ich versuche nicht zu viele Erwartungen an eine Person zu stellen. | Wording was propoesed to change. | Ich versuche, nicht zu viel von einer Person zu verlangen. |

**1.2 Supplementary Table S2: Cognitive debriefing queries and decisions from the expert-committee**

**1.2.1. Coping with pain**

| **Original Dutch Item** | **Pre-final German translation** | **Queries** | **Final German version** |
| --- | --- | --- | --- |
| 8. Ik onderbreek mijn bezigheden met rustpauzes. | Ich unterbreche meine Beschäftigungen mit Ruhepausen. | Regarding the word “ onderbreek“, the translation into “unterbrechen” is correct, but seems too literally and thus, was changed into “Ruhepausen einlegen”. Overall, the chosen translations of some items such as 8. | Ich lege Ruhepausen zwischen meinen Aktivitäten ein. |
| 10. Ik probeer niet bij de pijn stil te blijven staan. | Ich versuche mich nicht mit dem Schmerz zu beschäftigen. | Perceived redundancy with items 18 and 19. | Ich versuche, nicht an den Schmerz zu denken. |
| 18. Ik neem iets onder handen om de pijn te vergeten. | Ich unternehme etwas, um den Schmerz zu vergessen. | Perceived redundancy with items 10 and 19. | Ich unternehme etwas, um den Schmerz zu vergessen. |
| 19. Ik probeer niet aan de pijn te denken. | Ich versuche nicht an die Schmerzen zu denken | Perceived redundancy with items 18 and 10. | Ich versuche, nicht an den Schmerz zu denken. |

**1.2.2 Coping with limitations**

| **Original Dutch Item** | **Pre-final German translation** | **Queries** | **Final German version** |
| --- | --- | --- | --- |
| **Omgaan met beperkingen** | **Umgang mit Einschränkungen** |  |  |
| 2. Zwaar werk laat ik liggen. | Schwere Arbeit lasse ich liegen. | Statement from one patient, which did not alter the translation: I usually keep doing difficult and strenous activities, because I'm too proud to ask for help. | Schwere Arbeit lasse ich liegen. |
| 4. Ik probeer de positieve kanten van de situatie te zien. | Ich versuche, die positiven Seiten der Situation zu sehen. | Wording was changed. | Ich versuche, die Situation von der positiven Seite zu sehen. |
| 8. Wat ik moet doen, doe ik op een aangepaste manier. | Was ich zu tun habe, tue ich auf eine angepasste Weise. | Wording was changed. | Was ich zu tun habe, tue ich auf angepasste Weise. |
| 12. Ik denk na over manieren waarop ik dingen toch kan doen. | Ich überlege, wie ich die Dinge doch tun kann. | Perceived redundancy of items 12, 17, 19, 21 | Ich überlege, wie ich die Dinge doch tun kann. |
| 17. Ik zoek een manier om iets tóch te kunnen. | Ich suche nach einer Möglichkeit, doch etwas tun zu können. | Perceived redundancy of items 12, 17, 19, 21, although they did not alter the translation. | Ich suche nach einer Möglichkeit, doch etwas tun zu können. |
| 18. Ik doe mijn werk met tussenpozen. | Ich mache meine Arbeit mit Pausen. | Wording was changed. | Ich mache während meiner Arbeit Pausen. |
| 19. Ik bedenk nieuwe manieren om de zaken aan te pakken. | Ich denke mir neue Möglichkeiten aus, um die Dinge anzugehen. | Perceived redundancy of items 12, 17, 19, 21 | Ich denke mir neue Wege aus, um Dinge anzugehen. |
| 21. Als iets niet lukt probeer ik het op een andere manier. | Wenn etwas nicht funktioniert, versuche ich es auf andere Weise. | Perceived redundancy of items 12, 17, 19, 21, although they did not alter the translation. | Wenn etwas nicht funktioniert, versuche ich es auf andere Weise. |

**1.2.3. Coping with dependency**

| **Original Dutch Item** | **Pre-final German translation** | **Queries** | **Final German version** |
| --- | --- | --- | --- |
| **Omgaan met afhankelijkheid:** | **Umgang mit Abhängigkeit** |  |  |
| 2. Ik maak mezelf nuttig voor anderen. | Ich mache mich nützlich für andere. | Wording was changed. | Ich mache mich für andere nützlich. |
| 3. Ik probeer in mijn afhankelijkheid te berusten. | Ich versuche, mich mit meiner Abhängigkeit abzufinden. | Queries which did not alter the translations:  - Perceived redundancy of items 3, 5, 7 and 11,  - I don’t feel dependent at all and it does not fit to my personality and my life style. | Ich versuche, mich mit meiner Abhängigkeit abzufinden. |
| 5. Ik leg me neer bij mijn afhankelijkheid. | Ich finde mich mit meiner Abhängigkeit ab. | Query which did not alter the translations:  Perceived redundancy of items 3, 5, 7 and 11 | Ich finde mich mit meiner Abhängigkeit ab. |
| 6. Ik houd rekening met anderen. | Ich nehme Rücksicht auf andere. | Wording was changed. | Ich nehme auf andere Rücksicht. |
| 7. Ik probeer vrede met mijn afhankelijkheid te hebben. | Ich versuche mit meiner Abhängigkeit Frieden zu schließen. | Query which did not alter the translations:  Perceived redundancy of items 3, 5, 7 and 11 | Ich versuche, mit meiner Abhängigkeit Frieden zu schließen. |
| 10. Ik probeer anderen te ontzien. | Ich versuche, andere zu schonen. | Querry which did not alter the translations:  Perceived redundancy of items 10 and 13 | Ich versuche, andere zu schonen. |
| 11. Ik neem m'n afhankelijkheid op de koop toe. | Ich nehme meine Abhängigkeit an. | Querry which did not alter the translations:  Perceived redundancy of items 3, 5, 7 and 11 | Ich nehme meine Abhängigkeit an. |
| 13. Ik probeer niet teveel te vragen aan één persoon. | Ich versuche, nicht zu viel von einer Person zu verlangen. | Querry which did not alter the translations:  Perceived redundancy of items 10 and 13 | Ich versuche, nicht zu viel von einer Person zu verlangen. |

**Supplementary Table S3:** **Descriptive statistics of the cognitive debriefing results**

| **Queries per quality criterion** | **Comprehensibility** | **Understandability** | **Acceptability** | **Response option** | **Total queries per participant & domain** |
| --- | --- | --- | --- | --- | --- |
| **Domain: Pain, 25 items** | | | | | |
| **Participant no.: 1** | 2 | 0 | 0 | 0 | 2 |
| **2** | 0 | 0 | 0 | 0 | 0 |
| **3** | 0 | 1 | 0 | 0 | 1 |
| **4** | 1 | 2 | 0 | 0 | 3 |
| **5** | 2 | 1 | 0 | 0 | 3 |
| **6** | 1 | 0 | 0 | 0 | 1 |
| **7** | 0 | 0 | 0 | 0 | 0 |
| **8** | 0 | 1 | 0 | 0 | 1 |
| **9** | 1 | 1 | 0 | 0 | 2 |
| **10** | 1 | 0 | 0 | 0 | 1 |
| **Total queries domain: pain** | 8 (summe der queries aller TN) | 6 | 0 | 0 | 14 |
| **Queries per item (%)** | 32% (8 queries / 25 items) | 24% | 0% | 0% | - |
| **Average number of queries per participant** | 0,8 (8 queries / 10 participants) | 0,6 | 0 | 0 | - |
| **Domain: Limitations, 23 items** | | | | | |
| **Participant no.: 1** | 0 | 1 | 0 | 0 | 1 |
| **2** | 0 | 0 | 0 | 0 | 0 |
| **3** | 1 | 1 | 0 | 0 | 2 |
| **4** | 1 | 2 | 0 | 0 | 3 |
| **5** | 2 | 1 | 0 | 0 | 3 |
| **6** | 1 | 0 | 0 | 0 | 1 |
| **7** | 2 | 0 | 0 | 0 | 0 |
| **8** | 1 | 1 | 0 | 0 | 1 |
| **9** | 1 | 1 | 0 | 0 | 2 |
| **10** | 1 | 0 | 0 | 0 | 1 |
| **Total queries domain: limitations** | **10** | **7** | **0** | **0** | **17** |
| **Queries per item (%)** | **40,0%** | **30,4%** | **0%** | **0%** |  |
| **Average number of queries per participant** | **0,7** | **0,7** | **0** | **0** |  |
| **Domain: Dependency, 13 items** | | | | | |
| **Participant no.: 1** | 2 | 1 | 0 | 0 | 0 |
| **2** | 2 | 1 | 0 | 0 | 0 |
| **3** | 1 | 1 | 0 | 0 | 2 |
| **4** | 1 | 0 | 0 | 0 | 0 |
| **5** | 0 | 0 | 0 | 0 | 0 |
| **6** | 2 | 1 | 0 | 0 | 3 |
| **7** | 0 | 0 | 0 | 0 | 2 |
| **8** | 0 | 0 | 0 | 0 | 0 |
| **9** | 0 | 0 | 0 | 0 | 0 |
| **10** | 0 | 0 | 0 | 0 | 0 |
| **Total queries domain: dependency** | **8** | **4** | **0%** | **0%** | **7** |
| **Queries per item (%)** | **61,5%** | **30,8%** | **0** | **0** | **-** |
| **Average number of queries per participant** | **0,8** | **0,4** | **0** | **0** | **-** |

**2. Supplementary Figure S1: Final German CORS version**

**CORS - COping with Rheumatic Stressors –**

**Fragebogen zum Umgang mit axialer Spondyloarthritis**

Basierend auf dem CORS-Fragebogen zum Umgang mit rheumatoider Arthritis (Universiteit Nijmegen, 1992)

Patient-ID: ____________ Datum: _____________

**Umgang mit Schmerzen**

Menschen, die unter Schmerzen leiden, entwickeln verschiedene Methoden, um mit Schmerzen umzugehen. Auf den folgenden Seiten finden Sie eine Anzahl von Aussagen, die beschreiben, was Sie machen oder denken können, wenn Sie Schmerzen haben.

Wir bitten Sie, nach jeder Aussage anzugeben, wie oft Sie die beschriebene Verhaltensweise ausführen.

Wie oft führen Sie etwas aus?

Nach jeder Aussage stehen 4 Zahlen, womit Sie angeben können, wie oft Sie diese Verhaltensweisen ausführen.

1: selten oder niemals

2: ab und zu

3: oft

4: sehr oft

Beispiel: Wenn Sie ab und zu ein Bad nehmen, wenn Sie Schmerzen haben, dann machen Sie einen Kreis um die Zahl 2 neben der Aussage "Ich bade oder dusche".

|  | **Selten oder nie** | **Ab und zu** | **Oft** | **Sehr oft** |
| --- | --- | --- | --- | --- |
| Ich nehme ein Bad oder dusche | 1 | 2 | 3 | 4 |

Denken Sie nicht lange und intensiv über eine Frage nach, sondern nehmen Sie die erste Antwort, die Ihnen einfällt, denn es ist meistens die Beste. Sie müssen jedoch bei jeder Frage eine Zahl einkreisen.

**Was tun Sie, wenn Sie Schmerzen haben?**

|  | **Selten oder nie** | **Ab und zu** | **Oft** | **Sehr oft** |
| --- | --- | --- | --- | --- |
| 1. Ich denke, dass die Schmerzen wohl auszuhalten sind. | 1 | 2 | 3 | 4 |
|  |  |  |  |  |
| 2. Ich beschäftige mich mit anderen Dingen. | 1 | 2 | 3 | 4 |
|  |  |  |  |  |
| 3. Ich konzentriere mich auf etwas anderes. | 1 | 2 | 3 | 4 |
|  |  |  |  |  |
| 4. Ich denke, dass der Schmerz weniger wird. | 1 | 2 | 3 | 4 |
|  |  |  |  |  |
| 5. Ich beschränke mich auf einfachen Tätigkeiten. | 1 | 2 | 3 | 4 |
|  |  |  |  |  |
| 6. Ich denke an angenehme Dinge oder Ereignisse. | 1 | 2 | 3 | 4 |
|  |  |  |  |  |
| 7. Ich denke, dass die Schmerzen danach weniger wer- den. | 1 | 2 | 3 | 4 |
|  |  |  |  |  |
| 8. Ich lege Ruhepausen zwischen meinen Aktivitäten ein. | 1 | 2 | 3 | 4 |
|  |  |  |  |  |
| 9. Ich suche Gesellschaft. | 1 | 2 | 3 | 4 |
|  |  |  |  |  |
| 10. Ich versuche, mich nicht mit den Schmerzen zu beschäftigen. | 1 | 2 | 3 | 4 |
|  |  |  |  |  |
| 11. Ich mache meine Arbeit, wenn ich mich besser fühle. | 1 | 2 | 3 | 4 |
|  |  |  |  |  |
| 12. Ich gehe nach draußen. | 1 | 2 | 3 | 4 |
|  |  |  |  |  |
| 13. Ich denke mir, dass es anderen noch schlechter geht. | 1 | 2 | 3 | 4 |
|  |  |  |  |  |
| 14. Ich lenke mich mit Lesen, Musik, Fernsehen oder dergleichen ab. | 1 | 2 | 3 | 4 |
|  |  |  |  |  |
| 15. Ich mache etwas, das ich schön finde. | 1 | 2 | 3 | 4 |
|  |  |  |  |  |
| 16. Ich versuche, optimistisch zu bleiben. | 1 | 2 | 3 | 4 |
|  |  |  |  |  |
| 17. Ich versuche, mich körperlich nicht anzustrengen. | 1 | 2 | 3 | 4 |
|  |  |  |  |  |
| 18. Ich unternehme etwas, um den Schmerz zu vergessen. | 1 | 2 | 3 | 4 |
|  |  |  |  |  |
| 19. Ich versuche, nicht an die Schmerzen zu denken. | 1 | 2 | 3 | 4 |
|  |  |  |  |  |
| 20. Ich ruhe mich im Sitzen oder Liegen aus. | 1 | 2 | 3 | 4 |
|  |  |  |  |  |
| 21. Ich beschäftige mich mit meinen Hobbys. | 1 | 2 | 3 | 4 |
|  |  |  |  |  |
| 22. Ich versuche, trotz des Schmerzes zu genießen. | 1 | 2 | 3 | 4 |
|  |  |  |  |  |
| 23. Ich gehe zu Bett. | 1 | 2 | 3 | 4 |
|  |  |  |  |  |
| 24. Ich mache etwas, damit ich den Schmerz nicht fühle. | 1 | 2 | 3 | 4 |
|  |  |  |  |  |
| 25. Ich versuche, den Mut nicht zu verlieren. | 1 | 2 | 3 | 4 |

**Umgang mit Einschränkungen**

Menschen mit körperlichen Einschränkungen haben verschiedene Vorstellungen davon, wie sie mit Einschränkungen umgehen. Auf den folgenden Seiten finden Sie einige Möglichkeiten zum Umgang mit Einschränkungen. Wir bitten Sie, nach jeder Aussage anzugeben, wie oft Sie das beschriebene Verhalten zeigen. Sie können dies tun, indem Sie eine der folgenden Zahlen einkreisen.

1: selten oder nie

2: ab und zu

3: oft

4: sehr oft

Denken Sie nicht zu lange über jede Frage nach, sondern beantworten Sie jede Frage.

|  | **Selten oder nie** | **Ab und zu** | **Oft** | **Sehr oft** |
| --- | --- | --- | --- | --- |
| 1. Ich bin zufrieden mit dem, was ich tun kann. | 1 | 2 | 3 | 4 |
|  |  |  |  |  |
| 2. Schwere Arbeit lasse ich liegen. | 1 | 2 | 3 | 4 |
|  |  |  |  |  |
| 3. Ich suche nach neuen Aktivitäten. | 1 | 2 | 3 | 4 |
|  |  |  |  |  |
| 4. Ich versuche, die Situation von der positiven Seite zu sehen. | 1 | 2 | 3 | 4 |
|  |  |  |  |  |
| 5. Ich berücksichtige meine Einschränkungen. | 1 | 2 | 3 | 4 |
|  |  |  |  |  |
| 6. Ich suche nach Lösungen für meine Einschränkungen. | 1 | 2 | 3 | 4 |
|  |  |  |  |  |
| 7. Ich denke daran, dass es einigen Menschen noch schlechter geht. | 1 | 2 | 3 | 4 |
|  |  |  |  |  |
| 8. Was ich zu tun habe, tue ich auf eine angepasste Weise. | 1 | 2 | 3 | 4 |
|  |  |  |  |  |
| 9. Ich probiere alles aus. | 1 | 2 | 3 | 4 |
|  |  |  |  |  |
| 10. Ich versuche optimistisch zu bleiben. | 1 | 2 | 3 | 4 |
|  |  |  |  |  |
| 11. Ich nehme mir Zeit für meine Aktivitäten. | 1 | 2 | 3 | 4 |
|  |  |  |  |  |
| 12. Ich denke darüber nach, wie ich die Dinge trotzdem tun kann. | 1 | 2 | 3 | 4 |
|  |  |  |  |  |
| 13. Ich versuche, Vertrauen in die Zukunft zu haben. | 1 | 2 | 3 | 4 |
|  |  |  |  |  |
| 14. Ich denke mir, dass nicht unbedingt alle Arbeiten ab- geschlossen sein müssen. | 1 | 2 | 3 | 4 |
| 15. Ich denke darüber nach, wie ich meine Aktivitäten am besten planen kann. | 1 | 2 | 3 | 4 |
|  |  |  |  |  |
| 16. Ich verteile meine Aktivitäten über den Tag. | 1 | 2 | 3 | 4 |
|  |  |  |  |  |
| 17. Ich suche nach einer Möglichkeit, doch etwas tun zu  können. | 1 | 2 | 3 | 4 |
|  |  |  |  |  |
| 18. Ich mache Pausen während meiner Arbeit. | 1 | 2 | 3 | 4 |
|  |  |  |  |  |
| 19. Ich denke mir neue Wege aus, um Dinge anzugehen. | 1 | 2 | 3 | 4 |
|  |  |  |  |  |
| 20. Ich ruhe mich beizeiten aus. | 1 | 2 | 3 | 4 |
|  |  |  |  |  |
| 20. Wenn etwas nicht funktioniert, versuche ich es auf andere Weise. | 1 | 2 | 3 | 4 |
|  |  |  |  |  |
| 22. Ich beende meine Aktivitäten rechtzeitig. | 1 | 2 | 3 | 4 |
|  |  |  |  |  |
| 23. Die schwere Arbeit überlasse ich anderen. | 1 | 2 | 3 | 4 |

**Umgang mit Abhängigkeit**

Im Folgenden werden einige Möglichkeiten aufgezeigt, wie Menschen mit ihrer Abhängigkeit von anderen umgehen. Bitte geben Sie bei jeder Aussage an, wie oft Sie das beschriebene Verhalten ausführen. Dies können Sie tun, indem Sie eine der folgenden Zahlen einkreisen.

1: selten oder nie

2: ab und zu

3: oft

4: sehr oft

Auch hier gilt, dass Sie bitte nicht zu lange über jede Frage nachdenken.

|  | **Selten oder nie** | **Ab und zu** | **Oft** | **Sehr oft** |
| --- | --- | --- | --- | --- |
| 1. Ich verstehe, das es nun einmal nicht anders ist. | 1 | 2 | 3 | 4 |
|  |  |  |  |  |
| 2. Ich mache mich für andere nützlich. | 1 | 2 | 3 | 4 |
|  |  |  |  |  |
| 3. Ich versuche, mich mit meiner Abhängigkeit abzufin- den. | 1 | 2 | 3 | 4 |
|  |  |  |  |  |
| 4. Ich versuche, das Beste daraus zu machen. | 1 | 2 | 3 | 4 |
|  |  |  |  |  |
| 5. Ich finde mich mit meiner Abhängigkeit ab. | 1 | 2 | 3 | 4 |
|  |  |  |  |  |
| 6. Ich nehme Rücksicht auf andere. | 1 | 2 | 3 | 4 |
|  |  |  |  |  |
| 7. Ich versuche, mit meiner Abhängigkeit Frieden zu schließen. | 1 | 2 | 3 | 4 |
|  |  |  |  |  |
| 8. Ich passe mich anderen an. | 1 | 2 | 3 | 4 |
|  |  |  |  |  |
| 9. Ich versuche, mich mit meiner Abhängigkeit nicht unter Druck zu setzen. | 1 | 2 | 3 | 4 |
|  |  |  |  |  |
| 10. Ich versuche, andere zu schonen. | 1 | 2 | 3 | 4 |
|  |  |  |  |  |
| 11. Ich nehme meine Abhängigkeit an. | 1 | 2 | 3 | 4 |
|  |  |  |  |  |
| 12. Ich versuche, etwas zurückzugeben. | 1 | 2 | 3 | 4 |
|  |  |  |  |  |
| 13. Ich versuche, nicht zu viel von einer Person zu verlan-  gen. | 1 | 2 | 3 | 4 |

1. If this field is left blank, the translator offered a version without querries which was immediately accepted as the consensus version for backward translation German -> Dutch (right column). [↑](#footnote-ref-1)
